# FROM MUSEUM TRANSLATION TO AUTHENTIC MATERIALS FOR ENGLISH LANGUAGE EDUCATION: TRANSLATING JAVANESE ARCHAEOLOGICAL HERITAGE INTO ENGLISH THROUGH MUSEUM SONOBUDOYO'S BILINGUAL TEXTS

**DOI:** 10.12688/f1000research.188400.1

**Published:** 2026-08-12

**Authors:** Lenggahing Asri Dwi Eko Saputri, Qurrota A’yunin, Rizky Nugraha, Adrian Adrian, Ismi Windarti, Hira Maryana, Vanial Aulia Kurniawan, Krisna Hendro Setiono, Nasrah Nasrah

**Affiliations:** 1 Universitas Gadjah Mada, Yogyakarta, Special Region of Yogyakarta, Indonesia; 2 Universitas Pendidikan Indonesia, Bandung, West Java, Indonesia; 3 Universitas Negeri Yogyakarta, Yogyakarta, Special Region of Yogyakarta, Indonesia

**Keywords:** Bilingual Museum Text; Javanese Archaeological Heritage; Translation Strategies; Heritage Representation; English Language Education

## Abstract

Museum bilingual texts play an important role in communicating cultural heritage to international audiences while offering potential resources for English language education. However, limited research has integrated translation strategies, the representation of archaeological and historical meanings, and the pedagogical value of bilingual museum texts within a single analytical framework. This study investigates the English translation of Javanese archaeological heritage texts in Museum Sonobudoyo, Yogyakarta. Specifically, it examines (1) the translation strategies employed, (2) how archaeological and historical meanings are represented in the English translations, and (3) how the bilingual texts can be utilized as authentic materials for English language education. Adopting a qualitative case study design, the study analyzed bilingual Indonesian–English museum texts collected through document analysis and purposive sampling. The findings reveal three translation tendencies: resistance, negotiation, and a combination of both. Resistance generally produces strong representation by preserving culture-specific terminology, Negotiation tends to generate weak representation through descriptive adaptation, and the combined tendency results in partial representation by balancing cultural authenticity with readability. Furthermore, the bilingual museum texts demonstrate significant potential as authentic materials for English language education by integrating archaeological, historical, and cultural knowledge with real-world English communication. This study contributes an integrated framework linking translation strategies, heritage representation, and English language education, providing practical implications for museum translation, heritage communication, and authentic language learning.

## Introduction

Archaeological heritage is an important source of knowledge about human history, culture, and civilization. Ancient temples, inscriptions, artifacts, statues, and historical sites provide evidence of how people lived, believed, and interacted in the past ( Suvrathan, 2022). These remains help researchers reconstruct historical events and understand cultural development across different periods. Beyond academic value, archaeological heritage also strengthens cultural identity and promotes appreciation of shared human history ( Dadizadeh & Doğan, 2024; Khater & Faik, 2025). Many archaeological sites are recognised as valuable cultural assets that should be preserved for future generations. As international tourism and cultural exchange continue to grow, archaeological heritage has become increasingly accessible to global audiences. Therefore, communicating archaeological heritage effectively has become increasingly important.

One of the most important institutions for communicating archaeological heritage is the museum. Museums preserve, interpret, and present historical objects to the public through exhibitions, catalogues, labels, and information panels ( Lamptey, 2022). To reach both local and international visitors, many museums provide bilingual texts, typically in the local language and English. These texts explain the origin, function, historical context, and cultural significance of archaeological objects, helping visitors understand their value. Bilingual communication also supports cultural exchange by making local heritage accessible to people from different linguistic backgrounds ( Arllati, 2025). However, museum texts must do more than translate words because they should accurately convey historical and archaeological meanings while remaining clear and easy to understand.

The complexity of museum translation is especially apparent in Javanese archaeological texts, which contain historical, cultural, and religious concepts closely connected to ancient kingdoms and local traditions. Many terms, including the names of temples, inscriptions, rituals, and archaeological artefacts, have no direct equivalents in English. This creates a dilemma for translators, who must decide whether to preserve the original cultural expressions or adapt them for easier understanding by international visitors. Every translation choice has consequences for how readers perceive the historical background and cultural significance of the objects being described. Translation trategies may produce different interpretations of the same heritage item. Therefore, translation strategies are not only linguistic techniques but also important tools for representing archaeological and historical meanings accurately. Choosing appropriate strategies helps maintain authenticity while ensuring that museum texts remain accessible, informative, and meaningful for a global audience.

Issues related to the translation of Javanese archaeological heritage also have important implications for English language education. As educational spaces, museums provide bilingual texts that connect language learning with authentic archaeological, historical, and cultural content ( Fazzi, 2025). Unlike textbooks, these texts expose learners to real-world English used in heritage communication while fostering intercultural understanding. However, their educational value depends on translation quality. Translations that preserve archaeological and historical meanings while remaining comprehensible help learners develop linguistic competence, cultural literacy, and intercultural awareness. In contrast, oversimplified translations may reduce learners’ understanding of Javanese heritage. Despite their educational potential, bilingual museum texts remain underutilized as authentic learning materials. Therefore, examining how translation strategies shape the representation of archaeological and historical meanings is essential for evaluating the pedagogical value of museum texts and their contribution to authentic, content-based English language education.

Research on museum translation and heritage communication can be broadly classified into four major themes. First, museum translation strategies and quality assessment, which focuses on translation strategies, techniques, practices, and the assessment of translation quality in museum texts ( Afifah et al., 2024; Jiang, 2010; Manfredi, 2021; Nurpermadi et al., 2020; Rojo, 2018). Second, museum translation and heritage representation, which examines the representation of cultural heritage meanings through cultural translation, resemiotization, and multimodal translation ( Anderson & Lotman, 2018; Jiang, 2012; Liao, 2018b, 2023; Song, 2023). Third, museum communication and multilingual accessibility, which explores how bilingual and multilingual communication enhances the accessibility of museum information and visitors’ experiences through exhibition texts, audio guides, websites, and guided tours ( I’tishomah et al., 2025; Lazzeretti & Gatti, 2022; Liao & Bartie, 2022; Yalowitz et al., 2015; Yu & Hirzel, 2024). Fourth, museum translation for education and public engagement, which investigates the role of museum translation in education, language learning, public engagement, and heritage communication ( Ghazi, 2022; Lehman et al., 2018; Liao, 2018a; Xia et al., 2025; Yang & Mustafa, 2025). These themes demonstrate the interdisciplinary scope of museum translation research and provide a foundation for further studies that integrate translation strategies, archaeological and historical representation, and English language education.

Despite the growing body of research on museum translation and heritage communication, no study has comprehensively integrated translation strategies, the representation of archaeological and historical meanings, and English language education within a single analytical framework, particularly in the context of Javanese archaeological heritage. This gap limits understanding of how translation choices influence heritage representation and how bilingual museum texts can be utilized for language learning. The novelty of this study lies in integrating translation studies, representation theory, and English language education to examine Museum Sonobudoyo’s bilingual texts. Accordingly, this study addresses three research questions:

RQ1: What translation strategies are employed in translating Museum Sonobudoyo’s archaeological heritage texts into English?

RQ2: How are archaeological and historical meanings represented in the English translations?

RQ3: How can the translated texts be utilized as authentic materials for English language education?

## Theoretical framework

### Translation strategies as intercultural communication

Translation is widely understood as a form of intercultural communication that mediates meanings between different languages and cultures rather than merely transferring linguistic forms ( House, 2015; Katan & Taibi, 2021). In this process, translators act as cultural mediators who communicate culturally embedded concepts to target readers ( Bedeker & Feinauer, 2006). To achieve this goal, they employ translation strategies, defined as conscious procedures or decisions used to solve linguistic, cultural, and communicative problems encountered during translation ( Bardaji, 2009). These strategies enable translators to balance accuracy, readability, and cultural representation according to the translation context. The present study interprets translation strategies through two broad tendencies. Resistance, proposed by Venuti (1995), emphasizes preserving the linguistic and cultural distinctiveness of the source text to maintain its authenticity. In contrast, Negotiation, introduced by Eco (2013), views translation as a process of negotiating meaning between the source text, translator, and target readers, allowing cultural adaptation to enhance comprehension while maintaining essential meanings. Therefore, translation involves strategic decisions that reflect either a tendency to preserve the cultural identity of the source text or to negotiate meaning for the target audience.

### Representation of archaeological and historical meanings

Hall (1997) theory of representation views representation as the process of producing and communicating meaning through language, signs, and cultural practices rather than simply reflecting reality. From a constructionist perspective, meanings are socially constructed and shaped by the linguistic and cultural choices used to represent people, objects, and events. In translation, these choices influence how archaeological and historical meanings are conveyed to target readers. Drawing on Stuart Hall’s constructionist perspective, this study operationalizes representation into three analytical levels: (1) strong representation, where archaeological and historical meanings are largely preserved; (2) partial representation, where essential meanings are retained but certain cultural or historical elements are reduced or simplified; and (3) weak representation, where meanings become generalized or substantially altered, resulting in a loss of heritage significance. This analytical framework provides a systematic basis for evaluating the extent to which translation strategies preserve or reshape the representation of Javanese archaeological heritage in English.

### English language education through authentic materials, English for specific purposes, and content and language integrated learning

English language education increasingly emphasizes the use of authentic materials to bridge classroom learning and real-world communication. According to Gilmore (2007), authentic materials are texts created for genuine communicative purposes rather than for language teaching. Such materials expose learners to natural language, authentic discourse, and meaningful cultural contexts, enabling them to develop communicative competence alongside linguistic accuracy. They also encourage learners to interpret language in context, understand culture-specific meanings, and engage in critical thinking through real-world communication. In heritage education, bilingual museum texts represent authentic materials because they provide naturally occurring English while conveying archaeological, historical, and cultural knowledge. Their use enables learners to experience how English functions beyond the classroom and promotes cultural learning through meaningful interaction with authentic heritage content. Consequently, integrating museum bilingual texts into English language education enhances language proficiency, intercultural awareness, and learners’ understanding of Javanese archaeological heritage through authentic communicative experiences.

English language education also benefits from the principles of English for Specific Purposes (ESP), which emphasize teaching language according to learners’ academic or professional needs. According to Hutchinson and Waters (1987), ESP integrates language learning with discipline-specific content, allowing learners to acquire communicative competence within meaningful contexts. This principle aligns closely with Content and Language Integrated Learning (CLIL), in which language is learned through subject matter rather than in isolation ( Dalton-Puffer & Nikula, 2014). Museum bilingual texts therefore provide valuable resources for integrating English with archaeology, history, and cultural heritage, enabling learners to develop both language proficiency and disciplinary knowledge simultaneously. Through authentic museum texts, learners encounter specialized vocabulary, interpret historical and archaeological information, and strengthen intercultural communication skills. The combination of ESP and CLIL thus provides a pedagogical foundation for utilizing Museum Sonobudoyo’s bilingual texts as authentic instructional materials that connect English language learning with Javanese archaeological heritage and real-world heritage communication.

## Method

This study employed a qualitative approach to explore how archaeological and historical meanings are represented in the English translation of Javanese archaeological heritage texts. It adopted descriptive, comparative, and interpretative methods. The descriptive method was used to identify and describe the translation strategies found in the bilingual museum texts. The comparative method examined the relationship between the Javanese source texts (ST) and their English translations as target text (TT) to identify translation choices, meaning shifts, and the representation of culture-specific concepts. The interpretative method was applied to explain how these translation strategies influenced the representation of archaeological and historical meanings and to evaluate the potential of the translated texts as authentic materials for English language education. The study was designed as a qualitative case study, focusing on Museum Sonobudoyo as a single case because it preserves a rich collection of Javanese archaeological heritage and provides bilingual Javanese–English texts that support heritage communication for both local and international visitors.

The data consisted of Javanese–English bilingual texts displayed at Museum Sonobudoyo, Yogyakarta. Museum Sonobudoyo was selected because it is one of Indonesia’s leading museums for Javanese cultural and archaeological heritage and provides extensive bilingual texts for both local and international visitors. The data included exhibition labels, artifact descriptions, information panels, and explanatory narratives related to Javanese archaeological heritage. Purposive sampling was used to select texts containing archaeological terminology, historical references, and culture-specific concepts that present translation challenges. Data were collected through document analysis by photographing and transcribing the bilingual texts from museum exhibitions. The Javanese source texts and their English translations were then compiled into a parallel corpus for systematic comparison. This corpus served as the primary data source for examining translation strategies, analyzing the representation of archaeological and historical meanings, and exploring the potential of the translated texts as authentic materials for English language education. Its bilingual heritage texts provide a rich context for investigating the intersection of translation, heritage communication, and language learning. As shown in Figure 1, the research framework presents the analysis of bilingual museum texts.

**Figure 1.**
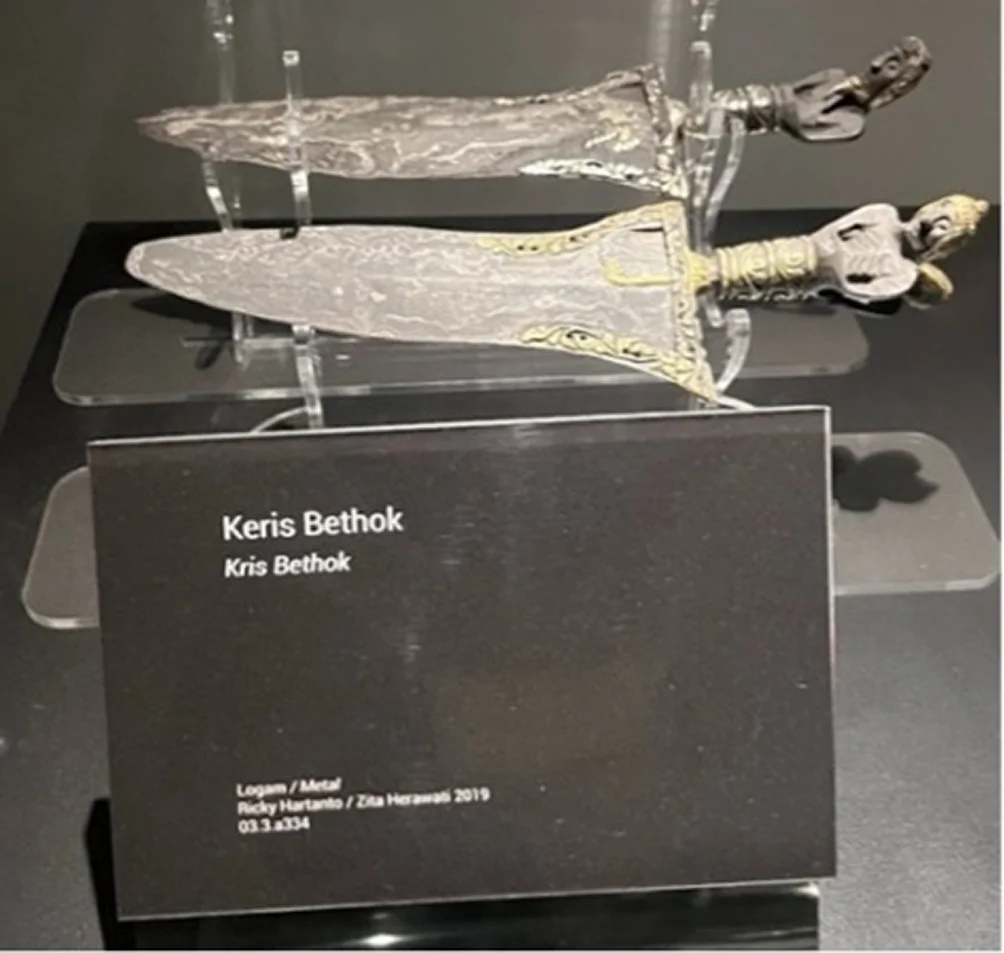
Example of a Bilingual text displayed in one of the collections at Museum Sonobudoyo. Source: Author’s documentation.

The data were analyzed by adapting the qualitative case study analysis model proposed by Yin (2016). The analysis consisted of five stages. First, compiling, in which all Javanese–English bilingual museum texts were collected and organized into a research corpus. Second, disassembling, where the corpus was segmented to identify translation units, archaeological and historical terms, and culture-specific elements. Third, reassembling, in which the identified data were systematically categorized according to translation strategies, representations of archaeological and historical meanings, and their potential educational functions. Fourth, interpreting, where the relationships between translation strategies and the representation of archaeological and historical meanings were examined, followed by an evaluation of how the translated texts could serve as authentic materials for English language education. Finally, concluding, the findings from all analytical stages were synthesized to establish the interrelationship among translation strategies, the representation of archaeological and historical heritage, and the educational potential of museum bilingual texts in supporting English language learning.

## Result

### Translation strategies in the English translation of javanese archaeological heritage texts

The analysis identified two broad translation tendencies employed in translating Javanese archaeological heritage texts into English, namely resistance and negotiation. These tendencies reflect the translators’ strategic decisions in balancing the preservation of source-culture identity with the communicative needs of target readers. By maintaining original lexical and cultural forms, this tendency seeks to retain the authenticity and distinctive identity of Javanese heritage while minimizing the loss of cultural meaning. The following section presents the findings related to the resistance tendency and explains how it is realized in the bilingual texts of Museum Sonobudoyo.

**Table:** 

(1)	ST:	*Di Jawa, dakon dimainkan anak-anak perempuan yang saling bergantian mengisi lubang menggunakan kuwuk (biji-bijian atau kulit kerang)*
	TT:	In Java, Dakon is played by girls who take turns filling the hole with kuwuk (seeds or shells)
(2)	ST:	*Di Jawa, dakon dimainkan anak-anak perempuan yang saling bergantian mengisi lubang menggunakan kuwuk (biji-bijian atau kulit kerang)*
	TT:	In Java, Dakon is played by girls who take turns filling the hole with kuwuk (seeds or shells)
(3)	ST:	*Permainan ini menggunakan sebuah papan berlubang dengan jumlah lubang beragam, ada yang 12, 16, hingga 20 lubang dengan dua buah lubang utama yang ada di sisi kiri kanan papan yang disebut lumbung gedhe*
	TT:	This game uses a board with a variety of holes, such as 12-hole, 16-hole, to 20-hole both with two main holes on the left and right side of the board that is called lumbung gedhe

The data (1), (2), and (3) demonstrate the application of the resistance tendency through the preservation of culture-specific lexical items in the target text. Instead of replacing Javanese cultural terms with English equivalents, the translator retains the original forms, such as *dakon*, *kuwuk*, and *lumbung gedhe.* This strategy reflects an intention to preserve the linguistic and cultural identity of the source text by exposing target readers to authentic Javanese terminology. In the first example, the term *dakon* is maintained rather than being translated as *mancala* or *traditional board game.* Likewise, *kuwuk* is retained, although a brief explanation— *seeds or shells*—is added in parentheses to facilitate comprehension. Similarly, *lumbung gedhe* remains untranslated, allowing the original cultural concept to be retained despite the absence of a direct English equivalent.

In addition to traditional games, the resistance tendency is also evident in the translation of archaeological objects associated with the everyday life of Javanese society. These culture-specific terms are preserved in their original forms rather than replaced with English equivalents. The following examples illustrate how this tendency is realized in the bilingual texts of Museum Sonobudoyo.

**Table:** 

(4)	ST:	*Kendi merupakan jenis gerabah yang terbuat dari tanah liat melalui proses pembakaran.*
	TT:	Kendi is a type of pottery made of clay through the firing proccess.
(5)	ST:	*Sepasang Lampu Robyong yang terbuat dari gelas dengan jumlah banyak. Berfungsi sebagai tempat penerangan. Hal ini dikaitkan dengan ilmu pengetahuan.*
	TT:	The pair of Lampu Robyong made of glass serves as a place of illumination and is associated with knowledge.
(6)	ST:	*Bokor digunakan sebagai pelengkap upacara*
	TT:	Bokor is used as a complement to the ceremonial ritual
(7)	ST:	*Kentongan atau slit drum merupakan alat komunikasi khusus pada masyarakat Jawa*
	TT:	Kentongan or slit drum is a special communication device of the Javanese communities

In data (4), the term *kendi* is retained and followed by a descriptive explanation, *a type of pottery made of clay through the firing process.* Instead of translating it as *water jug* or *pitcher*, the translator preserves the indigenous name while explaining its material. Similarly, data (5) retains *Lampu Robyong* and provides a functional description of its role as a lighting device associated with knowledge. In data (6), *bokor* is maintained in the target text, while its ceremonial function is explained without replacing the term with a generic equivalent such as *bowl* or *tray.* Likewise, data (7) preserves *kentongan* and adds the descriptive equivalent *slit drum*, enabling readers to understand its form while maintaining its original cultural identity.

The resistance tendency further extends to the translation of the components of Javanese *gamelan* instruments. As these terms represent culture-specific musical heritage with no direct English equivalents, they are retained in the target text to preserve their authenticity. The following examples demonstrate this translation strategy.

**Table:** 

(8)	ST:	*Bonang termasuk salah satu jenis instrumen gamelan yang berbentuk seperti gong*
	TT:	Bonang is one of Gamelan instruments which has a similar shape as a gong
(9)	ST:	*Kempyang merupakan instrumen sejenis simbal yakni alat musik yang berbentuk seperti mangkuk terbalik*
	TT:	Kempyang is a kind of cymbal, a music instrument that looks like an inversed bowl
(10)	ST:	*Kemanak merupakan salah satu instrumen gamelan yang berbentuk sangat khas karena menyerupai pisang*
	TT:	Kemanak is one of the gamelan instruments, that is shaped like a banana
(11)	ST:	*Wilahan atau bilah merupakan komponen instrumen gamelan yang berupa bilah-bilah berbentuk persegi panjang*
	TT:	Wilahan or slab, is a part of gamelan set, consisting of rectangular metal slabs

In data (8), *bonang* is retained and explained as one of the gamelan instruments with a shape similar to a gong. Likewise, data (9) preserves the term *kempyang*, while providing a descriptive explanation that it is a kind of cymbal resembling an inverted bowl. In data (10), *kemanak* remains untranslated and is accompanied by a description of its distinctive banana-like shape. Similarly, data (11) retains *wilahan* while adding the alternative term *slab* to describe its physical form as rectangular metal bars used in the gamelan.

Besides adopting the resistance tendency, the translator also applies negotiation in translating Javanese archaeological heritage texts. Through this tendency, culture-specific concepts are adapted or explained to enhance accessibility for international readers without completely abandoning their cultural significance. The following data demonstrate this translation tendency.

**Table:** 

(12)	ST:	*Ani-Ani *
	TT:	Paddy Harvesting Cutter
(13)	ST:	*Kalamudeng*
	TT:	Numerology Calendar
(14)	ST:	*Canthik*
	TT:	Boat Figurehead
(15)	ST:	*Pakinangan*
	TT:	Betel Nut Container
(16)	ST:	*Wayang*
	TT:	Puppet

In Data (12), *ani-ani* is translated as paddy harvesting cutter, emphasizing its agricultural function rather than retaining its indigenous name. Likewise, *kalamudeng* in Data (13) becomes numerology calendar, highlighting its cultural purpose in determining auspicious dates. In Data (14), *canthik* is rendered as boat figurehead, describing the carved ornament based on its location and function. Similarly, *pakinangan* in Data (15) is translated as betel nut container, making its practical use immediately understandable. In Data (16), *wayang* is translated as puppet, a familiar English term that facilitates comprehension for readers who may be unfamiliar with Javanese performing arts.

Beyond the resistance and negotiation tendencies, the analysis also identified cases in which both tendencies were employed simultaneously within the same translation unit. In these cases, the translator retains the original Javanese expressions to preserve their cultural identity while simultaneously rendering that align with the linguistic and communicative conventions of the target text. The following examples illustrate this integrated translation strategy.

**Table:** 

(17)	ST:	*Kain Batik Brangtamangu*
	TT:	Brangtamangu Batik
(18)	ST:	*Kain Batik Peksi Kurung*
	TT:	Peksi Kurung Batik
(19)	ST:	*Kain Batik Putri Piningit*
	TT:	Putri Piningit Batik
(20)	ST:	*Kain Batik Semen Sida Asih*
	TT:	Semen Sida Asih Batik
(21)	ST:	*Kain Batik Semen Rama*
	TT:	Semen Rama Batik
(22)	ST:	*Kain Batik Sida Mukti*
	TT:	Sida Mukti Batik
(23)	ST:	*Kain Lurik Lasem*
	TT:	Lasem Lurik
(24)	ST:	*Kain Batik Semen Cuwiri*
	TT:	Semen Cuwiri Batik
(25)	ST:	*Kain Batik Gringsing Buron Toya*
	TT:	Gringsing Buron Toya Batik
(26)	ST:	*Kain Batik Satriya Manah*
	TT:	Satriya Manah Batik
(27)	ST:	*Kain Batik Grompol*
	TT:	Grompol Batik

As shown in data (17)–(27), the lexical items *Brangtamangu*, *Peksi Kurung*, *Putri Piningit*, *Semen Sida Asih*, *Semen Rama*, *Sida Mukti*, *Lasem*, *Semen Cuwiri*, *Gringsing Buron Toya*, *Satriya Manah*, and *Grompol* are all preserved in their original forms. These motif names carry distinctive historical and cultural meanings and therefore remain untranslated to retain their authenticity. At the same time, the generic noun *kain batik* is reorganized into Batik, while *kain lurik* becomes Lurik, following English noun phrase conventions by placing the specific modifier before the generic noun (e.g., *Kain Batik Brangtamangu* → *Brangtamangu Batik*; *Kain Lurik Lasem* → *Lasem Lurik*).

The combined tendency is not limited to the translation of batik textiles but also appears in the translation of Javanese keris. The following data illustrate this combined translation tendency.

**Table:** 

(28)	ST:	*Keris Kalawijan*
	TT:	Kris Kalawijan
(29)	ST:	*Keris Pamengkang Jagad*
	TT:	Kris Pamengkang Jagad
(30)	ST:	*Keris Bethok*
	TT:	Kris Bethok

As shown in Data (28)–(30), the culture-specific names *Kalawijan*, *Pamengkang Jagad*, and *Bethok* are retained in their original forms, reflecting the resistance tendency. These names identify particular types of keris and embody historical and cultural values associated with Javanese weaponry. At the same time, the generic term *keris* is rendered as ‘kris’, representing the conventional English spelling that is more familiar to international readers. Rather than fully translating the artefacts into a generic expression such as *dagger*, the translator adopts the recognized English form while preserving the indigenous identity of each keris.

### Representation of archaeological and historical meanings in the English translations

The findings indicate that the representation of archaeological and historical meanings in the English translations can be classified into three levels: strong, partial, and weak representation. These levels reflect the extent to which the translations preserve the identity, cultural values, historical context, and heritage significance of Javanese archaeological objects. The level of representation is closely associated with the translation tendencies identified in the previous section. Resistance generally produces strong representation by preserving culture-specific terminology, negotiation tends to result in weak representation through descriptive adaptation, whereas the combination of both tendencies commonly generates partial representation by balancing cultural preservation with linguistic adaptation.

Strong representation is achieved when the translations preserve the original names of archaeological objects together with their cultural identity, historical significance, and functional characteristics. As illustrated in data (1)–(11), culture-specific terms such as *dakon*, *kuwuk*, *lumbung gedhe*, *kendi*, *Lampu Robyong*, *bokor*, *kentongan*, *bonang*, *kempyang*, *kemanak*, and *wilahan* are retained in the target text. Most of these terms are accompanied by descriptive explanations that clarify their functions, materials, or physical characteristics without replacing the original terminology. Consequently, target readers are introduced to authentic Javanese concepts while receiving sufficient contextual information to understand their archaeological and historical significance. This preservation of indigenous terminology enables the translations to maintain both the identity of the artefacts and their connection to Javanese cultural heritage.

Partial representation occurs when the translations preserve the core cultural identity of the artefacts but adapt certain linguistic elements to conform to English conventions. This pattern is evident in Data (17)–(30), where the names of batik motifs ( *Brangtamangu*, *Peksi Kurung*, *Semen Rama*, *Grompol* ) and keris ( *Kalawijan*, *Pamengkang Jagad*, *Bethok*) remain unchanged, while the generic nouns are adjusted to English forms, such as *Batik*, *Lurik*, and *Kris.* Although these translations successfully retain the unique identity of the artefacts, they provide limited information regarding their symbolic meanings, historical background, or cultural functions. As a result, readers can recognize the artefacts as elements of Javanese heritage but may not fully understand the cultural narratives associated with them. The translations therefore preserve heritage identity while only partially representing their broader archaeological and historical meanings.

Weak representation is found when culture-specific terminology is replaced by descriptive or functional English equivalents, resulting in the loss of indigenous identity. As shown in Data (12)–(16), *ani-ani *, *kalamudeng*, *canthik*, *pakinangan*, and *wayang* are translated as *paddy harvesting cutter*, *numerology calendar*, *boat figurehead*, *betel nut container*, and *puppet.* These translations effectively communicate the general functions of the artefacts, making them readily understandable for international readers. However, they largely omit the original Javanese terminology and the cultural, historical, and symbolic meanings embedded within these artefacts. Consequently, the translations reduce the uniqueness of Javanese heritage by presenting the objects as generic categories rather than culturally specific artefacts. This level of representation prioritizes communicative accessibility but substantially weakens the archaeological and historical identity conveyed in the source text.

### Museum bilingual texts as authentic materials for English language education

The findings indicate that the bilingual texts of Museum Sonobudoyo possess considerable potential as authentic materials for English language education. As naturally occurring texts produced for museum visitors rather than instructional purposes, they provide learners with exposure to real-world English used to communicate archaeological, historical, and cultural knowledge. This authenticity enables learners to engage with language in meaningful contexts while simultaneously developing an understanding of Javanese cultural heritage.

The analysis further shows that the bilingual texts present a wide range of authentic linguistic features, including culture-specific terminology, descriptive texts, explanatory discourse, and specialized vocabulary related to archaeology, history, and material culture. Examples such as *dakon*, *kendi*, *bonang*, *kentongan*, and *wayang* expose learners to lexical items that rarely appear in conventional English textbooks. At the same time, the accompanying English translations demonstrate how culturally embedded concepts can be communicated across languages using different translation strategies. Consequently, learners are introduced not only to new vocabulary but also to cross-cultural communication and intercultural meaning negotiation.

The findings also suggest that the three levels of representation identified in this study provide valuable pedagogical resources. Strong representation enables learners to recognize authentic Javanese terminology together with its archaeological and historical significance. Partial representation encourages learners to compare preserved cultural terms with adapted English expressions, promoting critical discussion of translation choices. Weak representation offers opportunities to evaluate the strengths and limitations of descriptive translations and to explore how cultural meanings may be reduced through adaptation. These variations can support translation analysis, vocabulary development, intercultural communication, and critical reading activities in English classrooms.

Furthermore, the bilingual texts are particularly relevant to English for Specific Purposes (ESP) because they introduce learners to English used in museum communication, heritage interpretation, tourism, and cultural promotion. Rather than learning isolated vocabulary or grammar, students encounter authentic discourse related to archaeology, history, and cultural heritage. Such materials can therefore support learners preparing for careers in museums, tourism, translation, archaeology, history, and other heritage-related professions where English is required for international communication.

Overall, the findings demonstrate that Museum Sonobudoyo’s bilingual texts extend beyond their communicative function as museum labels. They represent valuable authentic learning resources that integrate language learning with archaeological and historical knowledge while fostering intercultural awareness. Their pedagogical value is closely linked to the quality of translation, as translation strategies determine how accurately archaeological and historical meanings are represented and, consequently, how effectively these texts can be utilized in English language education.

## Discussion

Museum Sonobudoyo, located in Yogyakarta, is one of Indonesia’s leading museums dedicated to preserving and exhibiting Javanese cultural heritage through extensive collections of archaeological artefacts, historical objects, traditional arts, and ethnographic materials ( Octina et al., 2025; Telaumbanua & Fitriyanto, 2025). Its exhibitions feature Indonesian–English bilingual texts containing Javanese cultural terms that explain the historical background, archaeological significance, and cultural values of the displayed collections, making the museum an important site for both heritage preservation and intercultural communication. These bilingual texts provide authentic examples of how Javanese archaeological and historical knowledge is translated into English for international visitors. Consequently, Museum Sonobudoyo offers an appropriate setting for investigating translation strategies, the representation of archaeological and historical meanings, and the educational potential of bilingual museum texts. The museum therefore serves not only as a cultural institution preserving Javanese heritage but also as a valuable resource for English language education through authentic, culturally rich learning materials.

The findings demonstrate that the translation of Javanese archaeological heritage in Museum Sonobudoyo is characterized by three translation tendencies: resistance, negotiation, and their combination. These findings support the view that translation is an act of intercultural communication rather than a purely linguistic transfer ( Liddicoat, 2016). The identified tendencies correspond to the theoretical perspectives of resistance ( Venuti, 1995), which prioritizes preserving the cultural identity of the source text, and negotiation ( Eco, 2013), which emphasizes adapting meaning to the communicative conventions of the target text. The coexistence of these tendencies indicates that museum translators continuously balance authenticity and accessibility. Rather than consistently preserving or adapting cultural expressions, translators select strategies according to the communicative function of individual heritage terms. This finding reinforces argument that translation strategies are conscious problem-solving procedures shaped by linguistic, cultural, and communicative considerations instead of being fixed translation methods ( Mukminin et al., 2025; Mukminin & Sajarwa, 2025).

The findings further reveal that translation strategies directly influence the representation of archaeological and historical meanings. Terms translated through Resistance generally achieve strong representation, while negotiation tends to produce weak representation, and the combination of preserving and adapting usually results in partial representation. This relationship confirms Hall's (1997) constructionist perspective that meaning is constructed through representational choices rather than merely transferred between languages. Preserving indigenous terms such as *dakon*, *kendi*, and *bonang*maintains the cultural identity and historical significance of Javanese artefacts, whereas descriptive renderings such as *puppet* or *paddy harvesting cutter* successfully communicate basic meanings but reduce their cultural specificity. Therefore, translation does not simply convey information about archaeological objects but actively shapes how international audiences understand Javanese heritage. The findings extend Hall’s theory by operationalizing representation into measurable levels of archaeological and historical representation within bilingual museum translation.

Museum Sonobudoyo’s bilingual texts possess considerable pedagogical value beyond their primary function as sources of museum information. The interaction between translation strategies and representation levels positions these texts as authentic language resources that integrate archaeology, history, and culture within meaningful communicative contexts. Unlike conventional instructional materials, bilingual museum texts are produced for real museum visitors and therefore expose learners to naturally occurring English used to interpret Javanese cultural heritage. This characteristic is consistent with Gilmore's (2007) concept of authentic materials, which emphasizes the use of real-world texts to develop communicative competence and intercultural understanding. Furthermore, the specialized vocabulary related to archaeology, heritage, and museum communication reflects the principles of English for Specific Purposes (ESP) proposed by Hutchinson and Waters (1987), enabling learners to engage with discipline-specific discourse in authentic settings. Figure 2 illustrates how bilingual museum texts can be pedagogically utilized to support English language education.

**Figure 2.**
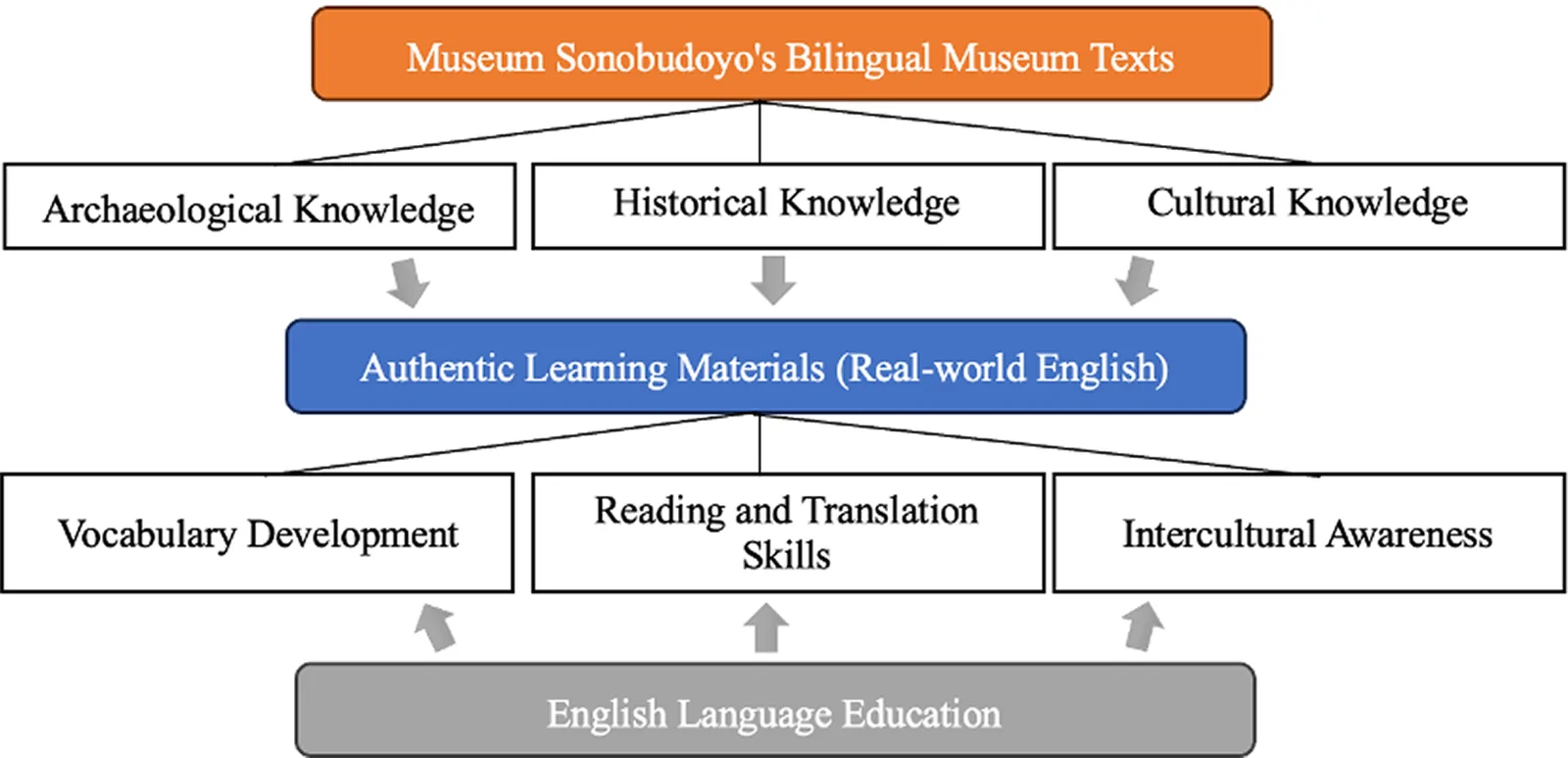
Pedagogical utilization of museum bilingual texts for English language education.

As illustrated in Figure 2, bilingual museum texts first provide learners with integrated archaeological, historical, and cultural knowledge before functioning as authentic learning materials. Through these authentic texts, learners encounter specialized vocabulary, descriptive discourse, and culture-specific concepts in realistic communication settings. The learning process subsequently contributes to vocabulary development, reading comprehension, translation skills, and intercultural awareness, enabling students to understand not only linguistic forms but also the cultural meanings embedded in Javanese heritage. This framework further demonstrates that the educational value of museum texts is closely associated with translation quality. Translations that preserve archaeological and historical meanings while remaining comprehensible provide richer learning resources than translations that excessively generalize cultural concepts. Consequently, bilingual museum texts can serve as interdisciplinary teaching materials that connect English language education with heritage education, intercultural communication, and cultural preservation, thereby expanding the role of museums as educational spaces beyond their traditional function as repositories of historical artefacts.

## Conclusion

This study demonstrates that the translation of Javanese archaeological heritage in Museum Sonobudoyo is characterized by three translation tendencies: resistance, negotiation, and a combination of both. These tendencies directly influence the representation of archaeological and historical meanings in the English translations. Resistance generally produces strong representation by preserving culture-specific terminology and cultural identity, whereas Negotiation tends to generate weak representation through descriptive adaptation that improves comprehensibility but reduces cultural specificity. The combined tendency results in partial representation, balancing cultural preservation with target-text conventions. The study further shows that bilingual museum texts have significant potential as authentic materials for English language education because they integrate archaeological, historical, and cultural knowledge within real-world English communication. By linking translation strategies, representation, and pedagogy in a single analytical framework, this study extends museum translation research beyond linguistic analysis and highlights the importance of producing bilingual museum texts that are both culturally authentic and pedagogically valuable for international communication and English language learning.

This study has several limitations. First, it focuses exclusively on the bilingual Javanese–English texts of Museum Sonobudoyo, limiting the generalizability of the findings to other museums or cultural contexts. Second, the analysis is confined to textual translation and does not examine visitors’ comprehension, reception, or learning outcomes resulting from the bilingual texts. Third, the study emphasizes translation strategies and representation without evaluating translation quality through expert assessment or user-based evaluation. Future research could address these limitations by investigating bilingual texts from multiple museums, comparing different types of cultural heritage, or incorporating multimodal elements such as images, exhibition design, and audio guides. Experimental or classroom-based studies are also recommended to examine the effectiveness of museum bilingual texts as authentic materials for English language education. Such research would provide stronger empirical evidence regarding the relationship between translation strategies, meaning representation, museum communication, and learning outcomes across diverse educational and cultural settings.

## AI declaration statement

During the preparation of this manuscript, AI-assisted tools were used exclusively to improve language quality, refine grammar, and assist with literature exploration and reference management. All research activities, including data collection, analysis, interpretation, and the development of arguments and conclusions, were carried out independently by the author. The author critically reviewed and verified all AI-assisted outputs and accepts full responsibility for the accuracy, integrity, and originality of the final manuscript.

## Data Availability

All data underlying the findings of this study are openly available in the Zenodo repository. The complete dataset analyzed during this study, including supporting research materials, is available at https://doi.org/10.5281/zenodo.21771362 ( Saputri et al., 2026). There are no restrictions on access to these data.
