# Supplementary material for: FROM MUSEUM TRANSLATION TO AUTHENTIC MATERIALS FOR ENGLISH LANGUAGE EDUCATION: TRANSLATING JAVANESE ARCHAEOLOGICAL HERITAGE INTO ENGLISH THROUGH MUSEUM SONOBUDOYO'S BILINGUAL TEXTS
Source: F1000Res. 2026 Aug 12;15:1356. [Version 1] doi: 10.12688/f1000research.188400.1 (PMC13646542; doi:10.12688/f1000research.188400.1)
Supplement: Peer review history

# Reviewer report: Reviewer response for version 1

**DOI:** 10.5256/f1000research.207928.r519756

**Published:** 2026-10-29

**Authors:** Novriyanto Napu

**Affiliations:** 1 Universitas Negeri Gorontalo, Gorontalo, Indonesia

The manuscript investigates the translation of Javanese archaeological and cultural heritage information into English through bilingual exhibition texts at Museum Sonobudoyo, Yogyakarta, Indonesia. It seeks to integrate three areas of inquiry: translation strategies, representation of archaeological and historical meanings, and the potential use of bilingual museum texts as authentic materials for English language education. 

 However, several methodological and interpretative issues require attention before the central findings can be considered sufficiently robust. The most important concerns involve the transparency of corpus selection, the operational definitions of translation and representation categories, the justification for associating particular translation strategies with specific levels of cultural representation, and the distinction between demonstrated findings and proposed educational applications.

 1. The Introduction organizes previous research into four themes: translation strategies and quality assessment, heritage representation, multilingual accessibility, and educational applications.

 Although this organization is useful, the discussion remains largely descriptive. It lists the areas addressed by previous studies without critically examining their methodological limitations, conflicting findings, or unresolved questions. 

 2. The authors should define archaeological heritage and clarify whether the study encompasses archaeological, historical, ethnographic, and intangible cultural heritage. The terminology should remain consistent throughout the manuscript.

 3. The manuscript repeatedly identifies the source texts as Javanese, although several examples are written in Indonesian and contain Javanese cultural expressions. The authors need to distinguish the linguistic identity of the source texts from the cultural identity of the objects being described.

 They should specify whether the corpus consists of Indonesian–English bilingual texts containing Javanese cultural terminology or whether some source texts are actually written in Javanese.

 The unit of analysis also requires clarification. It is not evident whether the study examines individual words, multiword expressions, complete sentences, exhibition labels, or entire descriptive panels. p.5-8

 4. Although purposive sampling is mentioned, the sampling procedures remain insufficiently documented.

 The authors should report how many bilingual texts were available, how many were collected, which were included in the final analysis, and why particular texts were selected. It is also important to explain whether the examples were selected because they illustrate the proposed translation categories or whether these categories emerged from examining the full dataset.

 The apparent duplication of examples (1) and (2) should be investigated. If these are genuinely separate museum labels containing identical text, they should be assigned distinct source identifiers. Otherwise, the duplication should be corrected.

 The authors should explain which museum collections were included and distinguish archaeological artifacts from ethnographic, artistic, and other cultural heritage objects where relevant. p.5

 5. The distinction between resistance and negotiation requires further theoretical clarification. Venuti's resistance-oriented perspective and Eco's understanding of translation as negotiation operate at different conceptual levels. Negotiation can occur even when a translator retains a culture-specific term. The concepts are therefore not necessarily mutually exclusive.

 The manuscript should explain why these theories were adopted as the principal classification framework and how the combination category differs analytically from its constituent tendencies. For example, retaining kendi while adding an English descriptive explanation could reasonably be interpreted as both cultural preservation and communicative adaptation. Yet the manuscript treats similar examples primarily as resistance. p.4-8.

 6. The manuscript appears to classify representation strength largely according to whether indigenous terms are preserved, modified, or replaced. Consequently, the reported association between translation strategies and representation levels may reflect the classification system itself rather than an independently demonstrated finding. For instance, translating wayang as puppet is classified as weak representation because the indigenous term is omitted. Although this English rendering may reduce cultural specificity, a judgment about historical or cultural meaning loss requires more evidence than lexical substitution alone.

 Similarly, translations retaining batik motif names are characterized as partial representation because detailed cultural meanings are not communicated. However, where the source text consists only of a motif name, the translation cannot necessarily be criticized for omitting information that was absent from the original.

 The authors should differentiate lexical preservation from semantic accuracy, historical fidelity, cultural specificity, and communicative accessibility.

 My suggestion is that the authors need to reassess the representation categories using independently defined criteria, demonstrate the reasoning through detailed source–target comparisons, and discuss examples that challenge the proposed associations. If necessary, revise the three-category relationship rather than treating it as a predetermined finding. p.9-10

 7. The manuscript does not adequately explain who performed the coding, whether interpretations were reviewed by multiple researchers, or how disagreements were resolved. Given the interpretative nature of the study, these details are important for assessing analytical trustworthiness.

 The authors should provide a coding framework illustrating the assignment of translation tendencies and representation levels. A useful supplementary matrix would link each source text and translation to its museum location, analytical category, justification, and any recorded ambiguity.

 The use of researcher triangulation, expert consultation, reflexive documentation, or another appropriate validation procedure should also be described where actually undertaken. p.5-6.

 8. The integration of museum translation and English language education is an interesting feature of the study. However, the analysis does not include a pedagogical intervention, classroom observations, learner assessments, or teacher evaluations.

 Consequently, claims about improvements in vocabulary development, reading comprehension, translation skills, and intercultural awareness remain theoretical propositions.

 Figure 2 provides a conceptual model of possible educational uses, but it should not be presented as evidence of demonstrated learning outcomes.

 The authors do not necessarily need to conduct a new experimental study. Given the exploratory design, they could strengthen this component by presenting specific instructional applications, such as comparative translation analysis, heritage vocabulary exercises, or critical interpretation tasks aligned with ESP or CLIL objectives.

 It would be useful if the authors also explicitly identify the educational framework as a proposed pedagogical application, present concrete examples of how the texts could be used, and avoid claiming demonstrated educational effectiveness. p.9-11.

 9. The analysis places considerable emphasis on whether culture-specific terminology is retained, but gives less attention to the linguistic accuracy and communicative quality of the English translations.

 Several reproduced target texts contain grammatical or lexical problems, including firing proccess and inversed bowl. These are particularly relevant because the article proposes using the translations as authentic instructional resources.

 The original museum texts should be reproduced faithfully, but their errors should not remain analytically unexamined. A text can preserve indigenous terminology while still being inaccurate, grammatically problematic, or difficult for international audiences to understand.

 Please include a separate consideration of linguistic accuracy and communicative clarity, identifying potentially problematic translations without altering the original source data. Explain whether such texts would require instructional annotation before classroom use. p.6-8.

 10. The interdisciplinary framing offers a potential contribution, but the novelty claim would be stronger if it identified a specific analytical or contextual advance rather than broadly asserting an absence of integrated studies.

 The limitations section recognizes the single-museum setting and lack of learner assessment. However, it should also acknowledge the possible subjectivity of classifying cultural representation, the dependence of findings on the proposed framework, and the limited basis for making claims about translation effects on actual audiences. p.3-4; 10-12.

Is the work clearly and accurately presented and does it cite the current literature?

Partly

If applicable, is the statistical analysis and its interpretation appropriate?

Not applicable

Are all the source data underlying the results available to ensure full reproducibility?

Partly

Is the study design appropriate and is the work technically sound?

Partly

Are the conclusions drawn adequately supported by the results?

Partly

Are sufficient details of methods and analysis provided to allow replication by others?

Partly

Reviewer Expertise:

Translation studies, applied linguistics (English).

I confirm that I have read this submission and believe that I have an appropriate level of expertise to state that I do not consider it to be of an acceptable scientific standard, for reasons outlined above.

# Reviewer report: Reviewer response for version 1

**DOI:** 10.5256/f1000research.207928.r522516

**Published:** 2026-10-07

**Authors:** Lalit Lalitav Mohakud

**Affiliations:** 1 Jadavpur University, Kolakata, West Bengal, India

**Review Report**

**Manuscript Title: **“ *From Museum Translation to Authentic Materials for English Language Education: Translating Javanese Archaeological Heritage into English Through Museum Sonobudoyo's Bilingual Texts*”

**Date:** 6 October 2026

**Summary of the Manuscript**

 The manuscript examines translation strategies, the representation of Javanese archaeological and historical meanings, and the pedagogical value of bilingual texts in Museum Sonobudoyo. A qualitative case study analysed Indonesian (Javanese)–English museum texts collected through document analysis. The findings reveal three translation strategies: resistance, negotiation, and a combination of both. Resistance produces a strong representation of culture-specific terminology; Negotiation reflects a weak representation through descriptive adaptation, and the combined tendency reflects a partial representation that balances cultural authenticity. The bilingual museum texts have great potential as authentic materials for English learning by combining archaeological, historical, and cultural knowledge with real-world English communication. The study presents an integrated framework that combines translation strategies, heritage representation, and English language education, offering practical implications for museum translation, heritage communication, and authentic language learning.

**Evaluation of the Manuscript**

 The manuscript addresses an important topic, and the use of a qualitative approach is appropriate for exploring the study's depth and complexity. The study demonstrates an integrated framework of bilingual translation strategies, representations of Javanese culture and English language education, which adds value to the existing body of knowledge. The presentation of the manuscript is well-written, well-justified, and clear, enhancing its authenticity and richness. The literature review is organised into four themes, which are well presented and justified. The theoretical framework, qualitative methodology, results, discussions, and conclusion are well-justified and nicely presented, enhancing the study's originality and its significant contribution to knowledge. However, minor revisions are needed; ‘Javanese cultural heritage’ and ‘Sonobudoyo Museum’ need explanation in the introduction section. 

***Recommendation: Approved***

 The manuscript addresses a relevant and potentially valuable research topic that could make a significant contribution to the field. Therefore, I recommend that the manuscript be approved for publication.

Is the work clearly and accurately presented and does it cite the current literature?

Yes

If applicable, is the statistical analysis and its interpretation appropriate?

Yes

Are all the source data underlying the results available to ensure full reproducibility?

Yes

Is the study design appropriate and is the work technically sound?

Yes

Are the conclusions drawn adequately supported by the results?

Yes

Are sufficient details of methods and analysis provided to allow replication by others?

Yes

Reviewer Expertise:

Educational Psychology, Cognitive Psychology, Tribal Studies, Creativity, Measurement and Evaluation in Education, Educational Technology.

I confirm that I have read this submission and believe that I have an appropriate level of expertise to confirm that it is of an acceptable scientific standard.

# Reviewer report: Reviewer response for version 1

**DOI:** 10.5256/f1000research.207928.r516200

**Published:** 2026-09-10

**Authors:** Allan Luke

**Affiliations:** 1 Queensland University of Technology, Brisbane, Australia

 Thanks for the opportunity to read and review this article, which takes on a topic of increasing significance in museum pedagogy, translation studies and, indeed, cultural and historical tourism. The focus here is on the accuracy, authenticity and revisionism in translation of Javanese concepts, ideas and perspectives into English for purposes of public pedagogy. 

 This text focuses primarily on the complexities of museum translation research, highlighting the interdisciplinary nature of the field. In short, the authors identify four principal themes which are instrumental to evaluating the effectiveness of translations. These themes are: the quality & strategy assessment of museum translations, effectiveness of cultural heritage representation, accessibility of multilingual communication techniques, and usefulness of translations in both a pedagogical and disseminative role. In particular, the analysis of this paper is based upon translation efforts conducted at the *Sonobudoyo Museum *in Yogyakarta, Indonesia. This museum contains the largest collection of Javanese archaeological and historical artefacts outside of Jakarta. Also of note is the popularity of the museum in an international tourism setting, hosting nightly Gamalan and Wayang performances. This context is ideally suited for analysis of translation effectiveness due to the necessity of English translations and the fact that many words in the Javanese language do not have English equivalents, prompting the need for careful and particular translation.

 The study concludes that at Sonobudoyo museum there exists three tendencies in English language translation. The first of these is resistance which involves the preservation of Javanese words in translations, with the intention of upholding and conveying cultural nuance and specificity (e.g. using the Indonesian word for water jug rather than ‘water jug’). The second is negotiation in which culture-specific terms are adapted and explained in translation. Words are generally negotiated if the specific uses of items can be more effectively and more immediately portrayed rather than through resistance measures. The third tendency involves a combination of both, an example being the use of English grammar conventions with Indonesian words (such as the possessive preceding the noun). This was primarily employed amongst items such as textiles where the origins and creators are especially important.

 In this way, the focus of this piece is a discourse analysis of the accuracy and, indeed, idiosyncrasy of translation. It is, in this regard, an example of critical textual analysis, if not a formal critical discourse analysis which features much more frequently now in critical translation studies more generally. As a 'first wave' study, this is adequate and well argued. What is missing are complementary foci that would logically follow any text analyses: (1) some documentation and analyses of the sources of the translation (e.g., the human linguistic agents and actors), and perhaps their trainings, intents, motivations and ideological presuppositions; (2) a much more fulsome study would be to engage with audience uptake - both expert, Indonesian and English museum visitors and tourists. This would indicate the actual degree to which the resistance, negotiation and combination strategies shape or mediate reader/viewer uptake of the text, but also the museum displays and overall experience. This said, the article is worth publishing as is; perhaps the authors might wish to outline an 'agenda for further research' in their conclusion.

Is the work clearly and accurately presented and does it cite the current literature?

Yes

If applicable, is the statistical analysis and its interpretation appropriate?

Not applicable

Are all the source data underlying the results available to ensure full reproducibility?

Yes

Is the study design appropriate and is the work technically sound?

Yes

Are the conclusions drawn adequately supported by the results?

Yes

Are sufficient details of methods and analysis provided to allow replication by others?

Yes

Reviewer Expertise:

literacy, language education, cultural anthropology and museum curation

I confirm that I have read this submission and believe that I have an appropriate level of expertise to confirm that it is of an acceptable scientific standard.
